# Hospital Meetings, &c.

**Published:** 1897-11-20

**Authors:** 


### HOSPITAL MEETINGS, &C.

### BRITISH HOME FOR INCURABLES.

The half-yearly election of candidates for admission to the Home for Incurables, Streatham, S.W., and for the pensions in connection with the Home, took place at Cannon Street Hotel on Tuesday, November 16th, 1897. At noon the Chairman, Mr. Francis Bevan, made a few introductory remarks. He stated that as the election was at the end of the half-year no report was issued. The society had made no special appeal for support, because of Her Majesty's festival of this year, but it was hoped that the heavy calls on behalf of other charities would not materially diminish subscriptions. About a month ago the funds were exhausted, but fortunately a timely gift of £1,000 came in from a lady, this followed by the promise of an annual subscription of 50 guineas; Lady Wallace also had left a legacy to the society of £5,000, which it was unfortunately feared would be subject to legacy duty both in England and France. Improvements have been made in the structure of the Home at Streatham; the top floor corridor has been widened and two additional rooms built, thus offering accommodation for 76 inmates. After the present election 72 beds will be occupied. The Home at Margate has been given up in consequence of the unsuitability of the house. A bungalow residence is the only kind that would enable incurables to take due advantage of their visit to the sea, and there is no money to build such a house. The money already subscribed for the purpose of the Margate Home is being used to send patients to convalescent homes, and a Samaritan Fund in connection with the society is badly wanted. On this occasion five candidates were admitted into the Home, and ten received a pension of £20 a year.

